# Intraoperative Methadone Versus Epidural Analgesia for Perioperative Pain Management in Major Abdominal and Thoracic Surgery: A Retrospective Single-Center Study

**DOI:** 10.3390/jcm15051696

**Published:** 2026-02-24

**Authors:** Arend Rahrisch, Sandra E. Guzzella, Samira Akbas, Julia Braun, Rolf Schüpbach, Donat R. Spahn, Alexander Kaserer

**Affiliations:** 1 Institute of Anesthesiology and Perioperative Medicine, University Hospital Zurich, University of Zurich, 8091 Zurich, Switzerland; arend.rahrisch@usz.ch (A.R.); sandra.guzzella@usz.ch (S.E.G.); samira.akbas@usz.ch (S.A.); rolf.schuepbach@usz.ch (R.S.); 2 Epidemiology, Biostatistics and Prevention Institute, University of Zurich, 8006 Zurich, Switzerland; julia.braun@uzh.ch; 3 Faculty of Medicine, University of Zurich, 8091 Zurich, Switzerland; donat.spahn@swisspbm.ch

**Keywords:** methadone, epidural, perioperative analgesia, postoperative pain, laparotomy, thoracotomy

## Abstract

**Background**: Adequate analgesia is essential for enhanced recovery following major abdominal and thoracic surgery. Intravenous methadone has emerged as an alternative analgesic modality to traditional epidural analgesia. This study compares intravenous methadone with epidural analgesia in postoperative pain. **Methods**: We retrospectively analyzed adult patients who underwent laparotomy or non-cardiac thoracotomy between January 2019 and December 2022 and who had either general anesthesia with epidural analgesia or intravenous methadone. Co-primary outcomes were mean numeric rating scale (NRS) pain scores and cumulative opioid consumption from extubation until the end of postoperative day 2. Pain scores were obtained regularly from routine postoperative assessments documented in the electronic health record and were not recorded at predefined postoperative hours. Secondary outcomes related to analgesia, recovery, and clinical outcomes were examined. **Results**: We analyzed 796 adults (mean age 58 ± 15 years, 52% male, 68% ASA III–IV), of which 691 (87%) underwent laparotomy and 105 (13%) underwent non-cardiac thoracotomy. Patients receiving methadone had a higher postoperative NRS score (0.4 points, 95% CI 0.23 to 0.62, *p* < 0.001), with a mean NRS of 2.1 ± 1.4 points in the methadone group and 1.6 ± 1.2 points in the epidural group. The postoperative opioid consumption (morphine equivalent dose) was lower in the methadone group (23 ± 31 vs. 29 ± 43 mg, −7.2 mg, 95% CI −12.6 to −1.79, *p* = 0.009). Methadone was associated with earlier mobilization (−0.13 days, 95% CI −0.24 to −0.01, *p* = 0.030). Epidural patients had greater need for escalation of laxatives (26% vs. 15%, *p* = 0.016), while time to extubation was shorter (8.4 min, 95% CI 6.2 to 10.5, *p* < 0.001). No differences were observed in maximum NRS, oxygen demand, blood product transfusions, major adverse cardiac and cerebrovascular events, or length of stay. **Conclusions**: Methadone was associated with higher, clinically non-relevant postoperative pain scores and a clinically non-relevant reduction of postoperative opioid use.

## 1. Introduction

Adequate analgesia following major abdominal and thoracic surgeries is essential for patients’ comfort, recovery and postoperative outcomes [1]. The insertion of an epidural catheter with continuous local anesthetic (epidural analgesia), often combined with opioids for postoperative analgesia, is considered the gold standard for major surgeries, such as laparotomies and thoracotomies. In open thoracotomy, procedure-specific recommendations highlight thoracic epidural analgesia or paravertebral blockade as first-line regional techniques, combined with baseline systemic analgesics [2]. Epidural analgesia (EDA) provides high-quality pain control, but its routine use can be limited by practical constraints, contraindications (e.g., coagulation disorders), and clinically relevant failure or discontinuation. Guidelines report clinically relevant epidural failure rates in the range of approximately 10–25%, and severe complications such as infection, epidural hematoma, and direct nerve trauma may occur, although rarely [3,4,5]. These limitations have contributed to increasing interest in alternative strategies that are feasible across heterogeneous surgical populations while maintaining effective analgesia.

Intraoperative intravenous (IV) methadone is an attractive option because of its long duration of action and its pharmacologic profile that may support sustained postoperative analgesia [6]. Randomized trials and meta-analyses suggest that a single intraoperative dose of methadone can reduce postoperative opioid requirements and may improve pain scores, and patient-reported satisfaction compared with shorter-acting opioid regimens [6,7,8,9,10]. At the University Hospital Zurich, IV methadone (0.2–0.3 mg kg^−1^) is therefore used as an alternative pain management when an EDA is not feasible. However, comparative effectiveness data versus epidural-based analgesia in routine care, particularly across major abdominal and thoracic surgery, remain limited. A pediatric study comparing multimodal analgesia with and without methadone to EDA found improved postoperative pain control, reduced opioid consumption, and shorter hospital stay in the methadone group [11]. The exclusive focus on children limits its applicability to adults.

We therefore compared intraoperative methadone with epidural analgesia in adults undergoing major laparotomy or non-cardiac thoracotomy, focusing on postoperative pain intensity and opioid requirements.

## 2. Materials and Methods

We conducted a retrospective single-center cohort study at University Hospital Zurich, Zurich, Switzerland, after obtaining approval of the local ethics committee (Cantonal Ethics Committee Zurich, Zurich, Switzerland, KEK-ZH-No.: 2023-00182, 21 March 2023). Data management followed Good Clinical Practice guidelines, and this manuscript adheres to the relevant Strengthening the Reporting of Observational Studies in Epidemiology guidelines.

### 2.1. Participants

We included adults (≥18 years) undergoing major laparotomy or non-cardiac thoracotomy between 1 January 2019 and 31 December 2022, receiving general anesthesia combined with either epidural analgesia or intraoperative IV methadone.

**Figure 1:**
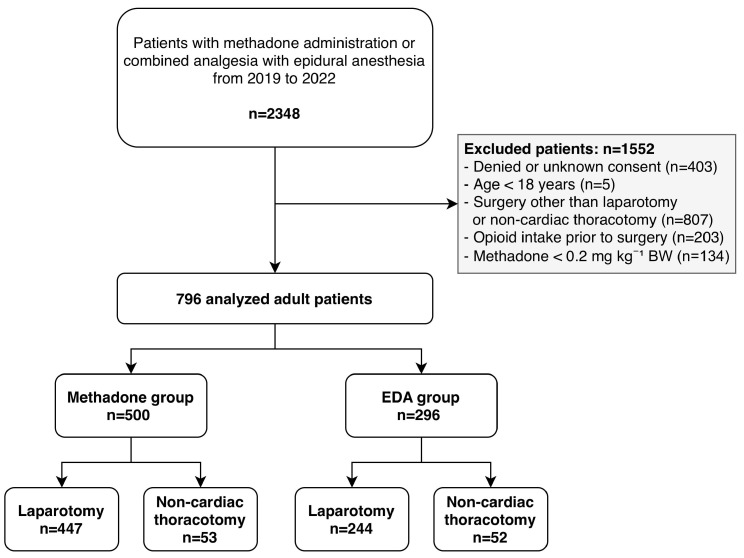
Flowchart of patient selection.

We excluded patients (i) receiving both epidural analgesia and intraoperative methadone, (ii) with daily opioid intake before hospital admission, (iii) requiring intraoperative extracorporeal life support or extracorporeal membrane oxygenation, (iv) not immediately extubated after surgery, and (v) denying consent for further use of their medical data. Patient selection is summarized in Figure 1.

### 2.2. Exposure

In the methadone group, 0.2–0.3 mg kg^−1^ ideal body weight [8,10] of methadone was administered during general anesthesia after induction but before incision.

In the EDA group, an epidural catheter at level corresponding to the planned surgery was placed prior to induction of general anesthesia. Epidural catheters were inserted preoperatively by experienced anesthesiologists, under the supervision of a consultant, using the loss-of-resistance technique. After identification of the epidural space, correct catheter position was assessed using a test dose of 3 mL lidocaine 2% with epinephrine 1:200,000 to exclude intrathecal or intravascular placement. Due to logistical and time constraints in routine care, the catheter was not fully loaded before induction of general anesthesia, and the sensory block level was therefore not systematically assessed pre-induction. Following induction, the epidural was titrated and the loading volume adjusted to patient height and the intended dermatomal spread to ensure adequate analgesia by the time of incision (ropivacaine 0.3%). After the initial loading dose, a continuous epidural infusion of ropivacaine 0.3% at 5–8 mL/h (height-adjusted) was commenced. Postoperatively, continuous epidural infusion was maintained with ropivacaine 0.2% plus fentanyl 2 µg/mL at an infusion rate of 5–8 mL/h, with patient-controlled boluses of 3 mL available once per hour. Sensory block extent was assessed at regular intervals and documented according to dermatomal distribution. In case of incorrect placement, the catheter was removed, and adequate alternative analgesia was established.

Further management of general anesthesia was at the discretion of the attending anesthesiologist and was predominantly provided as total intravenous anesthesia with propofol; when clinically indicated, inhalational anesthesia with sevoflurane was used. Postoperative nausea and vomiting (PONV) prophylaxis was administered according to local standards considering the baseline risk as assessed by the Apfel score [12]. Depending on surgical requirements, either deep neuromuscular blockade (post-tetanic count < 10) or moderate neuromuscular blockade (train-of-four count 0) was maintained, predominantly using rocuronium as the neuromuscular blocking agent.

### 2.3. Outcomes

This study primarily aimed to assess the analgesic efficacy of single IV methadone (0.2–0.3 mg kg^−1^) compared to an epidural catheter regimen with local anesthetic. The co-primary outcomes were the mean pain score on the numeric rating scale (NRS) and cumulative postoperative opioid consumption in morphine equivalents from the time of extubation through the end of postoperative day (POD) 2. Pain scores were obtained regularly from routine postoperative assessments documented in the electronic health record and were not recorded at predefined postoperative hours. As this was a retrospective study, pain assessments were performed according to clinical routine and patient needs rather than at predefined postoperative hours. Therefore, all available NRS measurements from extubation until the end of POD 2 were used to calculate the mean NRS. We used the NRS due to higher feasibility in routine care (including verbal administration) and because it is the standardized pain assessment tool used hospital-wide, ensuring consistent documentation. The NRS ranges in 11 steps from 0 (=no pain) to 10 (=worst imaginable pain) and was assessed at regular intervals during routine postoperative care both at rest and during movement, as standardized in our hospital [13]. Rescue opioids were considered by nurses and physicians when NRS exceeded 3. Morphine equivalents for fentanyl, oxycodone, and hydromorphone were calculated using the hospital’s standard opioid conversion tool (Opimeter) [14]. Neuraxial opioids delivered via epidural infusion/boluses were analyzed as part of the epidural technique and were not converted into systemic morphine equivalents.

Secondary outcomes were grouped into three domains to assess additional potential benefits or adverse effects: (1) analgesia-related outcomes, including the percentage of pain assessments indicating inadequate analgesia (NRS > 3) per patient, the maximum NRS pain score, and the type and total amount of postoperative analgesics (including non-opioid agents); (2) recovery outcomes, including time to extubation, postoperative need for oxygen supplementation (oxygen supplementation for more than six hours after extubation or an increase by ≥ 100% in patients with pre-existing oxygen therapy), duration of postoperative norepinephrine infusion, day of initial mobilization, and escalation of laxative therapy; and (3) clinical outcomes, including the number of blood products transfused, incidence of major adverse cardiac and cerebrovascular events (MACCE), length of hospital stay, and in-hospital mortality. MACCE was defined as a composite of postoperative myocardial infarction, cardiac arrest or cardiogenic shock, and cerebrovascular ischemic events occurring during the index hospitalization. Events were ascertained retrospectively from documented diagnoses in the electronic health record, including discharge summaries and relevant specialist reports.

Additional variables included the type and amount of analgesics administered (fentanyl, morphine, oxycodone, hydromorphone, metamizole, paracetamol, ibuprofen, diclofenac, ketamine), the administered dose of naloxone (opioid antagonist), and ondansetron (antiemetic).

### 2.4. Data Collection

We extracted data from digital anesthesia and health records of eligible patients, which were retrieved from the hospital’s clinical information systems (MetaVision^®^, iMDsoft^®^, Tel Aviv-Jaffa, Israel, and KISIM, Cistec AG, Zurich, Switzerland). All data analyzed were routinely collected as part of standard clinical care. The extracted data were subsequently organized in a spreadsheet (Excel 2016, Microsoft Corporation, Redmond, WA, USA) for analysis.

### 2.5. Statistical Analysis

For continuous data, we show mean values and their standard deviations. For categorical data, numbers and percentages are shown.

The two groups were compared using linear and logistic regression models, adjusted for the variables sex, age, American Society of Anesthesiologists (ASA) physical status class, and type of surgery. To analyze the percentage of measurements with inadequate analgesia per patient, a mixed logistic regression model was calculated for the binary variable denoting the presence of inadequate analgesia (NRS larger than 3). This model was adjusted for time since the end of anesthesia, sex and age, but not for ASA class and type of surgery. This was because quasi-complete separation would lead to numerical problems if more variables were included.

In a post hoc sensitivity analysis for a primary outcome, a linear mixed model for NRS over time was calculated, taking all measurements into account, using the same covariates as the models mentioned above, along with time since end of anesthesia and a random intercept per person. As an additional sensitivity analysis, all models were also estimated using inverse probability weighting, with propensity scores generated from the four variables included in the original adjustment: sex, age, ASA classification, and type of surgery.

The statistical analysis of the data was carried out in collaboration with the University of Zurich, Epidemiology, Biostatistics and Prevention Institute (EBPI), using RStudio (R version 4.5.0, Posit PBC, Boston, MA, USA). The significance level for primary analysis was 0.025 after Bonferroni adjustment for two primary outcomes. The significance level for the exploratory secondary analyses was 0.05.

### 2.6. Declaration of Generative AI and AI-Assisted Technologies in the Writing Process

During the preparation of this work the authors used ChatGPT-5.2 (OpenAI, San Francisco, CA, USA) for linguistic refinement. After using this tool, the authors reviewed and edited the content as needed and take full responsibility for the content of the publication.

## 3. Results

A total of 796 patients were included in the analysis, with 500 in the methadone group and 296 in the EDA group (Figure 1). Descriptive statistics revealed comparable demographics between the groups, with a mean age of 58 (±15) years in the methadone group and 58 (±14) years in the EDA group (Table 1). Data revealed comparable mean duration of anesthesia in both groups.

### 3.1. Primary Analysis

The mean NRS for pain was 2.1 ± 1.4 points in the methadone group and 1.6 ± 1.2 points in the EDA group. Multiple regression adjusted for sex, age, ASA physical status class, and type of surgery yielded a regression coefficient of 0.42 (95% CI: 0.23, 0.62, *p* < 0.0001) for methadone (Table 2). The amount of postoperative opioid analgesics, measured as the morphine equivalent dose, was lower in the methadone group with 23 ± 31 mg when compared to the EDA group with 29 ± 43 mg (−7.2 mg, 95% CI: −12.6 to −1.8, *p* = 0.009, Table 2).

### 3.2. Sensitivity Analysis

**Figure 2:**
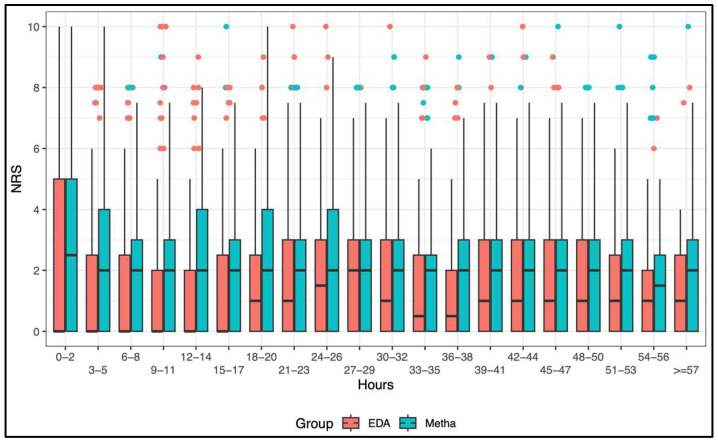
Boxplots for NRS values over time. EDA = epidural analgesia; Metha = methadone.

Considering all NRS measurements in a sensitivity analysis, a linear mixed model demonstrated an on average 0.42 higher NRS over time in the methadone group (0.42 points, 95% CI: 0.24 to 0.61, *p* < 0.001, Table 2 and Table S3). Figure 2 illustrates the trend of NRS values over time of both groups.

For an additional sensitivity analyses, a logistic regression model was used to generate the propensity scores included the four variables originally adjusted for sex, age, ASA classification, and type of surgery. The results of all models were very similar to our original models with adjustment.

### 3.3. Secondary Analyses

For analgesia secondary outcomes, mixed logistic regression models showed a higher percentage of measurements with inadequate analgesia (NRS > 3) in the methadone group (OR 1.73, 95% CI: 1.36 to 2.21, *p* < 0.001, Table S2). However, maximum NRS values did not differ (coefficient for methadone −0.08, 95% CI: −0.44 to 0.28, *p* = 0.661, Table 3).

Looking at recovery outcomes, regression analyses showed that the methadone group exhibited a longer time to extubation (8.4 min, 95% CI: 6.2 to 10.5, *p* < 0.001, Table 3). Time to initial mobilization was shorter in the methadone group (−0.13 days, 95% CI: −0.24 to −0.01, *p* = 0.030, Table 3). Logistic regression models showed a lower laxative use in the methadone group (OR 0.62, 95% CI: 0.42 to 0.92, *p* = 0.016, Table 3). Patients who underwent combined anesthesia with epidural analgesia exhibited a higher mean norepinephrine infusion time compared to those patients who underwent methadone treatment (9.4 ± 14.0 vs. 6.2 ± 15.8 h, Table S4).

For clinical secondary outcomes, no evidence for differences was observed for length of stay, prolonged oxygen supplementation or MACCE (Table 3). The amount of blood products transfused during primary hospital stay was comparable between both groups (Table 3).

The mean cumulative dose of postoperative ondansetron for antiemetic therapy was comparable between groups (3.8 ± 5.3 vs. 3.6 ± 5.6 mg, Table S1).

## 4. Discussion

Major surgical procedures require effective, multimodal strategies for perioperative pain control. While epidural analgesia remains the gold standard for large-incision surgery, its use is often limited by contraindications, technical challenges, or patient preference [3,4]. These limitations highlight the need for alternative opioid-based approaches that are both effective and feasible. Methadone has attracted attention as a potential candidate in this setting. A systematic review and meta-analysis by Machado et al. identified methadone as a preferred intraoperative opioid, demonstrating reduced postoperative pain scores for up to 48 h and improved patient satisfaction sustained for up to 72 h postoperatively [15].

**Figure 3:**
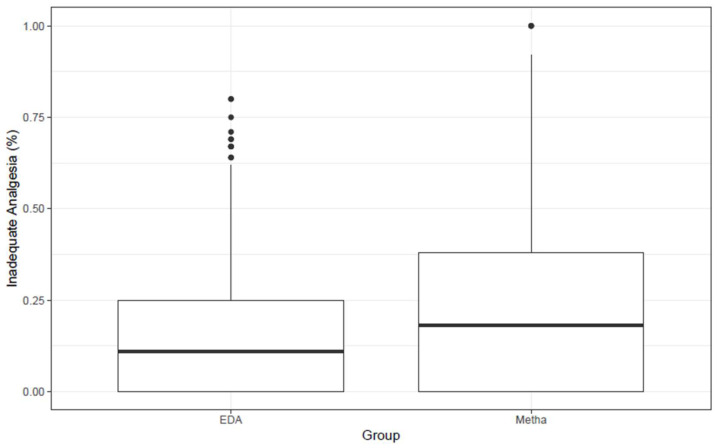
Boxplots of percentage of measurements with inadequate analgesia by group. EDA = epidural analgesia; Metha = methadone.

In our cohort, intraoperative IV methadone (0.2–0.3 mg kg^−1^) was associated with higher postoperative pain scores and a greater percentage of NRS > 3 measurements during POD 0–2 compared with EDA. However, maximum NRS values did not differ, and the incidence of inadequate analgesia was low and clustered near 0% in both groups (Figure 3). Although methadone was associated with a higher mean postoperative pain score compared with EDA (NRS mean difference 0.42 points, *p* < 0.001), this is unlikely to represent a clinically meaningful change in pain intensity, as a minimum difference of 1.0–1.3 points is generally required [16]. These data should be interpreted as routine-care comparative effectiveness, not as causal inference. Methadone’s clinical utility may relate to its prolonged duration of action and ease of intraoperative administration. Machado et al. reported the greatest analgesic benefit within 24–48-h postoperatively, aligning with our findings of notably low NRS scores in this timeframe (Figure 2) [15]. Similarly, Komen et al. demonstrated that even up to 30 days postoperatively, ambulatory surgical patients receiving intraoperative methadone used half as many opioid pills and reported significantly lower pain at rest [17]. The NRS is a pragmatic and widely used perioperative pain outcome because it is simple, can be administered verbally, and supports repeated assessment. Trials in common surgeries have used NRS to compare acetaminophen monotherapy with combinations (e.g., with pethidine or parecoxib), demonstrating its ability to detect clinically relevant differences in analgesic efficacy [18].

Postoperative opioid consumption was significantly lower in the methadone group, which may represent an advantage over EDA-based multimodal analgesia. Accordingly, Singh et al. reported reduced postoperative opioid needs and faster opioid weaning in coronary artery bypass grafting patients receiving intraoperative methadone compared to a non-methadone approach with ketamine and other opioids [19]. In a randomized trial in spine surgery, Murphy et al. also demonstrated reduced opioid use, lower pain scores, and improved patient satisfaction with methadone [10].

Furthermore, in our study, methadone was associated with an 8-min delay in extubation compared with EDA. While prior studies found no significant differences in extubation times between methadone and other opioid-based strategies [20,21,22], our findings align with Singh et al., who reported longer extubation times after methadone compared to non-methadone use in cardiac surgery [19]. Taken together, evidence regarding methadone’s effect on extubation timing is mixed. Given typical anesthetic durations of 7 h and extubation times of 20–30 min for major laparotomy and thoracotomy, we do not consider an 8-min difference to be clinically meaningful.

Earlier mobilization was observed in the methadone group compared with EDA, though the clinical relevance of the absolute 0.13-day difference remains uncertain. This may be particularly beneficial in enhanced recovery after surgery (ERAS) pathways by enhancing patient comfort, thereby facilitating earlier mobilization [10,15,23]. Although EDA has been associated with improved out-of-bed mobilization after colon surgery compared with morphine-based patient-controlled analgesia [24], it may also cause orthostatic hypotension, potentially delaying mobilization [25].

Methadone can dose-dependently affect respiratory function, including ventilation and oxygenation [26,27]. Despite concerns about respiratory depression and hypoxia, our study found no significant increase in postoperative oxygen supplementation in methadone-naive patients compared with those treated with EDA. While respiratory depression is a recognized effect of methadone, it is typically well managed in clinical practice [26,27]. In a pediatric study, children receiving IV methadone (0.1 mg kg^−1^) showed greater reductions in oxygen saturation than those given morphine or pethidine following ophthalmic surgery, yet none required intervention for hypoventilation or apnea [28]. Rapid redistribution lowers plasma methadone below the respiratory depression threshold (approximately 100 ng/mL) within 45 min after bolus [27,29]. Well-anticipated dosing—particularly in combination with other opioids—and close postoperative respiratory monitoring are recommended.

Gastrointestinal paralysis is common after abdominal surgery [30]. In our study, methadone patients required significantly fewer escalations in laxative therapy compared with EDA. This contrasts with a systematic review by Guay et al., in which EDA demonstrated improved postoperative bowel function versus IV opioid-based analgesia (excluding methadone), reflected by shorter times to first flatus and stool [31]. We observed reduced opioid use following intraoperative methadone, which may contribute to the reduction of opioid-induced bowel dysfunction and reduced need for laxative escalation.

We observed a non-significant ~threefold higher MACCE rate in the methadone group than with EDA. Importantly, four events occurred >7 days after methadone administration, beyond its expected pharmacologic effect (half-life 8–59 h, up to 120 h) [32,33,34]. Although methadone can prolong QT and rarely cause torsade de pointes [32,33,35], this is predominantly reported with chronic or high-dose exposure, and evidence for methadone-associated cardiac events remains limited [33]. Overall, the timing and clinical heterogeneity of events suggest underlying comorbidity rather than a direct methadone effect. Evidence on neuraxial techniques and cardiac outcomes is mixed: guidelines mention EDA for analgesia and potential cardiac risk reduction [36], whereas a POISE post hoc analysis reported increased MI, non-fatal cardiac arrest, and cardiovascular death with neuraxial blockade [37]. EDA was also associated with longer postoperative norepinephrine infusion (9.4 vs. 6.2 h), consistent with sympathetic blockade and vasodilation-related hypotension [38,39,40,41,42,43,44]. Further studies should clarify whether methadone offers hemodynamic advantages over EDA without increasing cardiovascular events.

Transfusion rates for erythrocyte concentrates, platelet concentrates, and fresh frozen plasma did not differ between the groups. While a prior study has linked EDA to increased transfusion rates [45], data specifically on methadone remain limited.

Hospital stay was non-significantly shorter in the EDA group compared with the methadone group. This contrasts with Boesl et al., who reported reduced length of stay with a multimodal approach combining methadone and wound infiltration versus EDA in cytoreductive surgery with hyperthermic intraperitoneal chemotherapy [46].

In the methadone group, eight in-hospital deaths occurred (1.6%) versus none in the EDA group. Five deaths followed transition to palliative best supportive care due to poor prognosis from underlying conditions (myocardial tumor infiltration, septic shock, two cases of liver failure with septic/hemorrhagic shock, and renal failure with sepsis). Three deaths resulted from sudden cardiac arrest (two obstructive shock, one with heparin-induced thrombocytopenia type II; one presumed myocardial syncope). Due to the limited number of events, no statistical comparison between groups was feasible. Reported postoperative in-hospital mortality ranges from 1.4–4.5% for elective laparotomy [47,48] and 1.5–7.5% for elective non-cardiac thoracotomy [49,50]. Accordingly, our cohort’s rate lies at the lower end. We found no evidence that intraoperative methadone increased in-hospital mortality compared with EDA.

Postoperative nausea and vomiting (PONV) was routinely managed with ondansetron as first-line therapy [51], with comparable cumulative doses in both groups, suggesting no relevant difference in PONV treatment between methadone and EDA.

From an economic perspective, single-dose IV methadone is substantially less costly than EDA. A standard dose (2 × 10 mg ampoules) costs 4 Swiss francs and is administered in a single step. In contrast, EDA incurs baseline material costs of 75 Swiss francs (including epidural kit, syringe pump kit, infusion line, ropivacaine 0.3%), requires continuous delivery via syringe pump, and daily postoperative assessments by trained physicians. These cumulative demands in equipment, staffing, and monitoring contribute to substantially higher overall costs associated with EDA.

## 5. Limitations

This study has several limitations. First, its retrospective, single-center design introduces the possibility of selection bias in patient allocation to specific analgesic strategies. Confounding by indication is likely, as epidurals are preferentially used in selected patients without contraindications. This is reflected by unequal group sizes, with the methadone group (n = 500) being nearly twice the size of the EDA group (n = 296). Without randomization, residual confounding may persist despite statistical adjustment for measured covariates; unmeasured differences in baseline characteristics influencing treatment allocation could have biased the observed associations. Moreover, the single-center setting and adherence to institution-specific protocols may limit generalizability. Because analgesic titration was part of routine care, our retrospective data do not capture the clinical rationale behind rescue opioid administration (e.g., concerns about sedation or respiratory depression). Therefore, we cannot determine whether differences in opioid titration practices may have contributed to the small between-group differences in NRS. Moreover, NRS may be subject to floor effects and reduced sensitivity at low pain levels. As pain scores were generally low in our cohort, small between-group differences should be interpreted with caution. Additionally, intraoperative anesthetic management cannot be reliably separated from the exposure groups, residual confounding related to intraoperative co-analgesics and anesthetic technique may have influenced postoperative outcomes. Finally, the low incidence of rare outcomes such as MACCE and mortality limited the statistical power to detect meaningful differences in these safety endpoints. Safety outcomes were exploratory; the study is not powered to detect differences in rare adverse events.

## 6. Conclusions

In this retrospective cohort, intraoperative methadone and epidural analgesia achieved overall low postoperative pain scores, suggesting broadly comparable clinical analgesia. Although methadone was associated with a statistically higher mean NRS during POD 0–2, the absolute difference was small and unlikely to be clinically meaningful, with inadequate analgesia remaining uncommon in both groups. Methadone was associated with a statistically significant but clinically non-relevant reduction in postoperative opioid use.

## Figures and Tables

**Table 1:** Patients undergoing major laparotomy or non-cardiac thoracotomy (n = 796).

		EDA Group n = 296	Methadone Group n = 500
Age (years)		58 (±14)	58 (±15)
Sex (male)		136 (46%)	275 (55%)
Height (cm)		170 (±9)	170 (±10)
Body mass index (kg/m^2^)		25.5 (±5.2)	26.4 (±5.6)
ASA class	1	2 (0.7%)	6 (1.2%)
	2	124 (41.9%)	127 (25.4%)
	3	161 (54%)	337 (67%)
	4	9 (3%)	30 (6%)
Duration of anaesthesia (hours)		7.1 (±2.4)	7.1 (±2.0)
Laparotomy		244 (82.4%)	447 (89.4%)
Non-cardiac thoracotomy		52 (17.6%)	53 (10.6%)

Data are presented as count and percentage (%), mean with standard deviation (±SD). ASA = American Society of Anesthesiologists.

**Table 2:** Primary outcomes with sensitivity analysis.

	EDA n = 296	Methadone n = 500	Regression Coefficient (95% CI)	*p*-Value
Primary outcomes				
Pain (NRS)	1.6 (±1.2)	2.1 (±1.4)	0.42 (0.23, 0.62) *	<0.001
	1.4 [0.7–2.3]	2 [1–3]		
Morphine equivalent dose (mg)	29 (±43)	23 (±31)	−7.20 (−12.60, −1.79) *	0.009
Sensitivity analysis				
NRS over time			0.42 (0.24, 0.61) ^†^	<0.001

Data are presented as mean with standard deviation (±SD) and median [IQR]. EDA = epidural analgesia, NRS = Numeric rating scale. * Linear models for continuous outcomes (reference category: epidural analgesia), adjusted for sex, age, ASA physical status class, and type of surgery. ^†^ Linear mixed model for NRS over time (reference category of group variable: epidural analgesia), adjusted for time since end of anesthesia, sex, age, ASA physical status class, and type of surgery.

**Table 3:** Secondary outcomes.

	EDA n = 296	Methadone n = 500	Odds Ratio or Regression Coefficient (95% CI)	*p*-Value
Analgesia-related outcomes (selected; additional in Table S1)				
Percentage of inadequate analgesia (NRS > 3) per patient	0.16 (±0.18)	0.23 (±0.24)	1.06 (0.76, 1.48) ^†^	0.727
Maximum NRS	4.7 (±2.5)	4.7 (±2.4)	−0.08 (−0.44, 0.28) *	0.661
	5 [3.9–7]	5 [3–6]		
Recovery outcomes				
Time to extubation (minutes)	18 (±12)	27 (±16)	8.35 (6.23, 10.47) *	<0.001
Postoperative day of initial mobilization	1.1 (±0.7)	1.0 (±0.8)	−0.13 (−0.24, −0.01) *	0.030
Oxygen supplementation > 6 h (yes)	154 (52%)	285 (57%)	1.18 (0.86, 1.61) ^†^	0.314
Escalation of laxatives (yes)	78 (26%)	77 (15%)	0.62 (0.42, 0.92) ^†^	0.016
Norepinephrine infusion (hours)	9.4 (±14.0)	6.2 (±15.8)		
Clinical outcomes				
RBC (units)	0.2 (±1.9)	0.5 (±2.0)	0.21 (−0.08, 0.51) *	0.162
FFP (units)	0	0.1 (±1.0)	0.05 (−0.07, 0.17) *	0.395
PC (units)	0.02 (±0.3)	0.03 (±0.3)	0 (−0.05, 0.05) *	0.980
MACCE	1 (0.3%)	7 (1.4%)	3.05 (0.50, 59.01) ^†^	0.311
Length of stay	12.3 (±10.7)	13.6 (±12.1)	0.75 (−0.98, 2.48) *	0.395
Death	0 (0.0%)	8 (1.6%)		

Data are presented as count and percentage (%), mean with standard deviation (±SD) and median [IQR]. EDA = epidural analgesia, NRS = Numeric rating scale, RBC = Red blood cell concentrate, FFP = Fresh frozen plasma, PC = Platelet concentrate, MACCE = Major adverse cardiac and cerebrovascular events. * Regression coefficient from linear regression models for continuous outcomes (reference category: epidural analgesia), adjusted for sex, age, ASA physical status class, and type of surgery. ^†^ Odds ratio from logistic regression models for binary outcomes (reference category: epidural analgesia), adjusted for sex, age, ASA physical status class, and type of surgery.

## Data Availability

The data presented in this study are available on request from the corresponding author due to ethical and data protection considerations.

## Supplementary Materials

The following supporting information can be downloaded at: https://www.mdpi.com/article/10.3390/jcm15051696/s1, Table S1: Additional outcomes for patients undergoing major laparotomy or non-cardiac thoracotomy (n = 796); Table S2: Results from the mixed logistic regression model for inadequate analgesia (reference category of group variable: epidural analgesia), adjusted for time since end of anesthesia, sex and age; Table S3: Results from the linear mixed model for NRS over time (reference category of group variable: epidural analgesia), adjusted for time since end of anesthesia, sex, age, ASA class and type of surgery; Table S4: Relation between duration of postoperative norepinephrine-therapy and MACCE; Figure S1: Numeric Rating Scale [52].

## Abbreviations

The following abbreviations are used in this manuscript:

ASA	American Society of Anesthesiologists
EDA	epidural analgesia
IV	intravenous
MACCE	major adverse cardiac and cerebrovascular events
NRS	numeric rating scale
POD	postoperative day
PONV	postoperative nausea and vomiting
