# Supplementary material for: Intraoperative Methadone Versus Epidural Analgesia for Perioperative Pain Management in Major Abdominal and Thoracic Surgery: A Retrospective Single-Center Study
Source: J Clin Med. 2026 Feb 24;15(5):1696. doi: 10.3390/jcm15051696 (PMC12986146; doi:10.3390/jcm15051696)

Supplementary Material

Content of additional tables:

- **Supplementary Table S1:** Additional outcomes for patients undergoing major laparotomy or non-cardiac thoracotomy (n=796).

- **Supplementary Table S2:** Results from the mixed logistic regression model for inadequate analgesia (reference category of group variable: epidural analgesia), adjusted for time since end of anesthesia, sex and age.

- **Supplementary Table S3:** Results from the linear mixed model for NRS over time (reference category of group variable: epidural analgesia), adjusted for time since end of anesthesia, sex, age, ASA class and type of surgery.

- **Supplementary Table S4:** Relation between duration of postoperative norepinephrine-therapy and MACCE.

- **Supplementary Figure S1:** Numeric Rating Scale by Jensen et al. Pain 1993;55: 195–203.

Table S1. Additional outcomes for patients undergoing major laparotomy or non-cardiac thoracotomy (n=796).

		EDA Group n=296	Methadone Group n=500
Ideal body weight (kg)		63.3 (± 9.6)	64.1 (± 10.6)
**Intraoperative analgesics**			
Methadone total (mg)		0 (± 0)	19.1 (± 9.2)
Methadone IBW (mg/kg IBW)		0 (± 0)	0.3 (± 0.2)
Ropivacaine total (mg)		241 (± 298)	0 (± 0)
Ropivacaine per hour (mg/h)		35.4 (± 47.6)	0 (± 0)
**Analgesia-related outcomes**			
**Postoperative analgesics**			
Paracetamol (mg)		6410 (± 3190)	5940 (± 3480)
Metamizole (mg)		7270 (± 2900)	7400 (± 2750)
Ibuprofen (mg)		15.5 (± 136)	10.6 (± 133)
Diclofenac (mg)		9.4 (± 40.1)	7.2 (± 39.0)
Ketamine (yes)		10 (3.4%)	35 (7.0%)
Morphine (mg)		4.9 (± 17.3)	8.9 (± 19.2)
Hydromorphone (mg)		0.3 (± 2.0)	0.4 (± 2.0)
Hydromorphone MED (mg)		2.3 (± 15.5)	2.5 (± 14.3)
Oxycodone (mg)		9.1 (± 21.8)	13.1 (± 20.1)
Oxycodone MED (mg)		4.5 (± 10.9)	6.6 (± 10.0)
Fentanyl (mcg)		176 (± 324)	46 (± 144)
Fentanyl MED (mg)		17.6 (± 32.4)	4.6 (± 14.4)
**Co-medication**			
Naloxone (mg)		1.7 (± 4.6)	2.3 (± 6.0)
Ondansetron (mg)		3.6 (± 5.6)	3.8 (± 5.3)
**Discipline**			
General/ Visceral		155 (52.4%)	408 (81.6%)
Thoracic		48 (16.2%)	48 (9.6%)
Gynecology		60 (20.3%)	24 (4.8%)
Urology		24 (8.1%)	16 (3.2%)
Plastics		9 (3.0%)	3 (0.6%)
Vascular		0 (0%)	1 (0.2%)
**Type of surgery**			
Urogenital		64 (21.6%)	39 (7.8%)
Thoracic		52 (17.6%)	53 (10.6%)
Gastrointestinal		131 (44.3%)	269 (53.8%)
Liver		49 (16.6%)	139 (27.8%)
**Type of surgery (detailed)**			
Urogenital	Uterus	40 (13.5%)	20 (4.0%)
	Urinary tract	3 (1.0%)	0 (0%)
	Kidney	13 (4.4%)	10 (2.0%)
	Prostate	2 (0.7%)	1 (0.2%)
	Bladder	4 (1.4%)	6 (1.2%)
	Adrenal	2 (0.7%)	2 (0.4%)
Thoracic	Thorax	47 (15.9%)	49 (9.8%)
	Esophagus	5 (1.7%)	4 (0.8%)
Gastrointestinal	Colon	13 (4.4%)	83 (16.6%)
	Stomach	12 (4.1%)	43 (8.6%)
	Retroperitoneal	6 (2.0%)	6 (1.2%)
	Cytoreductive	27 (9.1%)	14 (2.8%)
	Spleen	0 (0%)	1 (0.2%)

	Hernia	73 (24.7%)	122 (24.4%)
Liver	Liver	25 (8.4%)	99 (19.8%)
	Pancreas	24 (8.1%)	40 (8.0%)

Data are presented as count and percentage (%), mean with standard deviation (± SD). IBW = Ideal body weight; MED = Morphine equivalent dose.

Table S2. Results from the mixed logistic regression model for inadequate analgesia (reference category of group variable: epidural analgesia), adjusted for time since end of anesthesia, sex and age.

	OR	CI, lower	CI, upper	p-value
Intercept	0.47	0.28	0.79	0.004
Group: Methadone	1.73	1.36	2.21	< 0.001
Time (hours)	0.99	0.98	0.99	< 0.001
Sex	1.16	0.92	1.46	0.215
Age	0.98	0.97	0.99	< 0.001

Data are presented as odds ratios with lower and upper 95% confidence interval and p-value.

Table S3. Results from the linear mixed model for NRS over time (reference category of group variable: epidural analgesia), adjusted for time since end of anesthesia, sex, age, ASA class and type of surgery.

	Coefficient	CI, lower	CI, upper	p-value
Intercept	2.56	1.61	3.52	< 0.001
Group: Methadone	0.42	0.24	0.61	< 0.001
Time (hours)	-0.01	-0.01	0	< 0.001
Sex	0.16	-0.02	0.35	0.082
Age	-0.02	-0.03	-0.02	< 0.001
ASA: 2	0.29	-0.60	1.18	0.524
ASA: 3	0.38	-0.51	1.28	0.401
ASA: 4	0.41	-0.57	1.38	0.418
Type: Thorax	-0.07	-0.43	0.29	0.697
Type: Gastro	-0.02	-0.30	0.26	0.884
Type: Liver	0.32	0.01	0.64	0.045

Data are presented as coefficient with lower and upper 95% confidence interval and p-value. ASA = American Society of Anesthesiologists classification; Type = Type of surgery.

Table S4. Relation between duration of postoperative norepinephrine-therapy and MACCE.

**NE time vs. MACCE**				
		No (N=788)	Yes (N=8)	Overall (N=796)
NE time (hours)	Mean	6.9 (± 14.1)	49.5 (± 44.3)	7.4 (± 15.2)
	Median	0 [0, 140]	46.4 [0, 146]	0 [0, 146]
**NE time vs. MACCE, EDA group only**				
		No (N=295)	Yes (N=1)	Overall (N=296)
NE time (hours)	Mean	9.3 (± 13.8)	54.0 (NA)	9.4 (± 14.0)
	Median	2.0 [0, 86.0]	54.0 [54.0, 54.0]	2.3 [0, 86.0]
**NE time vs. MACCE, Methadone group only**				
		No (N=493)	Yes (N=7)	Overall (N=500)
NE time (hours)	Mean	5.5 (± 14.1)	48.9 (± 47.8)	6.2 (± 15.8)
	Median	0 [0, 140]	43.0 [0, 146]	0 [0, 146]

Data are presented as mean with standard deviation (± SD) and median (Min, Max). NE time = Norepinephrine infusion time.

Figure S1: Numeric Rating Scale by Jensen et al. Pain 1993;55: 195–203.

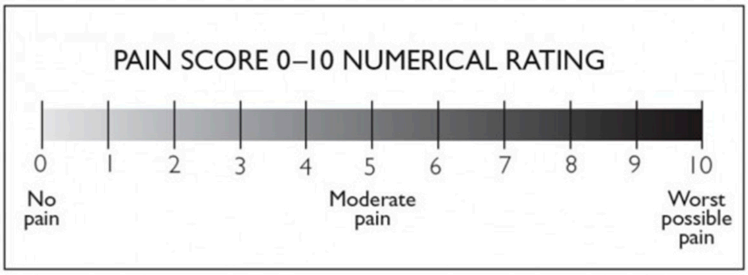

Supplement: Supplementary file 1 [file jcm-15-01696-s001.zip › jcm-4139674-supplementary.pdf]
